# Enhanced Sensitivity of Nonsmall Cell Lung Cancer with Acquired Resistance to Epidermal Growth Factor Receptor-Tyrosine Kinase Inhibitors to Phenformin: The Roles of a Metabolic Shift to Oxidative Phosphorylation and Redox Balance

**DOI:** 10.1155/2021/5428364

**Published:** 2021-07-29

**Authors:** Suntae Kim, Ji Hye Im, Wan Kyu Kim, Young Jae Choi, Ji-Yoon Lee, Sang Kyum Kim, Sun Jo Kim, Sung Won Kwon, Keon Wook Kang

**Affiliations:** ^1^College of Pharmacy and Research Institute of Pharmaceutical Sciences, Seoul National University, Seoul 08826, Republic of Korea; ^2^Department of Cancer Control, National Cancer Center Graduate School of Cancer Science and Policy, National Cancer Center, Goyang 10408, Republic of Korea; ^3^Department of Life Sciences, Ewha Womans University, Seoul 03760, Republic of Korea; ^4^College of Pharmacy, Chungnam National University, Daejeon 34134, Republic of Korea; ^5^Bioanalysis and Pharmacokinetics Research Group, Korea Institute of Toxicology, Daejeon 34114, Republic of Korea

## Abstract

**Background:**

Although the efficacy of epidermal growth factor receptor-tyrosine kinase inhibitor (EGFR- TKI) therapy has been proven in non-small cell lung cancer (NSCLC) patients, acquired resistance to EGFR-TKIs presents a serious clinical problem. Hence, the identification of new therapeutic strategy is needed to treat EGFR-TKI-resistant NSCLC.

**Methods:**

Acquired EGFR-TKI-resistant lung cancer cell lines (HCC827, H1993, and H292 cells with acquired resistance to gefitinib or erlotinib) were used for cell-based studies. IncuCyte live cell analysis system and XFp analyzer were used for the determination of cell proliferation and energy metabolism, respectively. *In vivo* anticancer effect of phenformin was assessed in xenografts implanting HCC827 and gefitinib-resistant HCC827 (HCC827 GR) cells.

**Results:**

HCC827 GR and erlotinib-resistant H1993 (H1993 ER) cells exhibited different metabolic properties compared with their respective parental cells, HCC827, and H1993. In EGFR-TKI-resistant NSCLC cells, glycolysis markers including the glucose consumption rate, intracellular lactate level, and extracellular acidification rate were decreased; however, mitochondrial oxidative phosphorylation (OXPHOS) markers including mitochondria-driven ATP production, mitochondrial membrane potential, and maximal OXPHOS capacity were increased. Cell proliferation and tumor growth were strongly inhibited by biguanide phenformin via targeting of mitochondrial OXPHOS complex 1 in EGFR-TKI-resistant NSCLC cells. Inhibition of OXPHOS resulted in a reduced NAD^+^/NADH ratio and intracellular aspartate levels. Recovery of glycolysis by hexokinase 2 overexpression in erlotinib-resistant H292 (H292 ER) cells significantly reduced the anticancer effects of phenformin.

**Conclusion:**

Long-term treatment with EGFR-TKIs causes reactivation of mitochondrial metabolism, resulting in vulnerability to OXPHOS inhibitor such as phenformin. We propose a new therapeutic option for NSCLC with acquired EGFR-TKI resistance that focuses on cancer metabolism.

## 1. Background

Epidermal growth factor receptor-tyrosine kinase inhibitors (EGFR-TKIs) such as gefitinib and erlotinib have generally been used in non-small cell lung cancer (NSCLC) patients as first-line targeted therapy since 2003, and their prognosis has significantly improved with the targeted therapy. Unfortunately, long-term treatment with EGFR-TKIs frequently causes resistance to targeted therapy, which led to the development of second- and third-generation EGFR-TKIs that avoid point mutations in the EGFR tyrosine kinase domain [1]. The T790M point mutation in EGFR exon 20 was reported as a major cause of EGFR-TKI resistance [1, 2]. Consequently, diverse resistance research was conducted on the H1975 NSCLC cell line carrying the T790M mutation, and a third-generation EGFR-TKI, osimertinib (Tagrisso®), which targets T790M, has been approved by the United States Food and Drug Administration as first-line therapy for metastatic lung cancer patients with an EGFR exon 19 deletion or L858R mutation [3]. However, acquired resistance to EGFR-TKIs may not be solely due to point mutations in some patients [2]. Although several molecular mechanisms for acquired resistance to EGFR-TKIs have been suggested, clinically available new therapeutic strategies are still needed [1].

Because a principal hallmark of cancer cells is rapid growth, a sufficient supply of nutrients is a critical requirement for cancer cells. Therefore, understanding cell type-specific metabolic processes that consume or use nutrients in cancer is important and could offer potential target(s) for cancer chemotherapy. After the discovery of the Warburg effect (metabolic dependency on glycolysis even under aerobic conditions), metabolic reprogramming in tumors has been extensively studied, and several clinically effective targets for controlling cancer cell-specific metabolism have been identified [4, 5]. In the present study, a new therapeutic strategy for treating EGFR-TKI-resistant NSCLC that focuses on cancer metabolism is proposed and the pharmacological efficacy of phenformin, a biguanide agent was elucidated.

## 2. Materials and Methods

### 2.1. Reagents and Antibodies

Antibodies recognizing Hexokinase I (C35C4), Hexokinase II (C64G5), phospho-pyruvate kinase isozymes M2 (PKM2) (Tyr105), PKM2 (D78A4), *α*-tubulin, phospho-EGFR (Tyr1068) (1H12), phospho-AKT (Ser473), and phospho-p44/42 mitogen-activated protein kinase (MAPK) (Thr202/Tyr204) (20G11) were purchased from Cell signaling Technology (Danvers, MA, USA). Anti-Pyruvate Dehydrogenase E1-alpha subunit (S293) and total OXPHOS human antibody cocktail were obtained from Abcam (Cambridge, United Kingdom). Antiglyceraldehyde-3-phosphate dehydrogenase (GAPDH) was supplied from Millipore (Burlington, MA, USA) and anti-Flag was purchased from Sigma Aldrich (St. Louis, MO, USA). Epidermal growth factor (EGF), sodium 2-oxobutyrate (AKB), and L-aspartic acid were supplied from Sigma Aldrich. Gefitinib and osimertinib were obtained from MedChemExpress (Monmouth Junction, NJ, USA). Erlotinib, phenformin, and rotenone were purchased from Cayman (Ann Arbor, MI, USA).

### 2.2. Cell Culture and Establishment of Stably Hexokinase II Overexpressing H292 ER Cells

Human lung cancer cells H1993, H1993 ER, H292, and H292 ER cells were kind gifts of Dr. Sang Kook Lee (Seoul National University, Seoul, Republic of Korea). HCC827, HCC827 GR, H1975, H1993, H1993 ER, H292, and H292 ER cells were cultured in RPMI 1640 medium (Hyclone, Logan, UT, USA) with 10% fetal bovine serum (Biowest, MO, USA) and 1% penicillin/streptomycin (100 U/mL, Hyclone). All cells were maintained in an incubator humidified 5% CO_2_ at 37°C.

To establish hexokinase II stably overexpressing cells, pCAG-Flag-HK2-IRES-Blas plasmid was transfected to H292 ER cells using Lipofectamine 2000 as specified by the manufacturer's instruction (Invitrogen, Carlsbad, CA, USA). Colonies were selected by incubation with blasticidin (15 *μ*g/mL, Invitrogen). pCAG-Flag-IRES-Blas vector was used for mock transfection. pCAG-Flag-IRES-Blas and pCAG-Flag-HK2-IRES-Blas plasmids were kindly donated from Dr. Hong-Duk Youn (Department of Molecular Medicine & Biopharmaceutical Sciences, Graduate School of Convergence Science, Seoul National University, Seoul, Republic of Korea).

### 2.3. Cell Proliferation

Cells were seeded in a 96-well plate (2 × 10^3^ cells/well) and cultured for 72 h. To determine cell proliferation, real-time scanned phase-contrast images were acquired and integrated confluence was analyzed by IncuCyte® ZOOM™ Live Cell Analysis system (Essen BioScience, Ann Arbor, MI, USA).

### 2.4. Genomic DNA Sequencing

Genomic DNA from lung cancer cell lines was extracted with lysis buffer (10% sodium dodecyl sulfate (SDS), 100 mM NaCl, 100 mM ethylenediaminetetraacetic acid (EDTA), 50 mM Tris (pH 8.0)) and proteinase K (15 mg/mL). Exon 18-21 region of EGFR gene was amplified by polymerase-chain-reaction (PCR). Thermal cycler settings include an initial denaturation at 95°C for 3 min followed by 40 cycles of denaturation at 95°C for 20 sec, annealing at 59.1°C for 10 sec, and extension at 72°C for 30 sec. Primers for exon 18 (sense: CAAATGAGCTGGCAAGTGCCGTGTC, antisense: GAGTTTCCCAAACACTCAGTGAAAC), exon 19 (sense: GCAATATCAGCCTTAGGTGCGGCTC, antisense: CATAGAAAGTGAACATTTAGGATGTG), exon 20 (sense: CCATGAGTACGTATTTTGAAACTC, antisense: CATATCCCCATGGCAAACTCTTGC), and exon 21 (sense: CTAACGTTCGCCAGCCATAAGTCC, antisense: GCTGCGAGCTCACCCAGAATGTCTGG) were used, and capillary electrophoresis sequencing was performed in Macrogen (Seoul, South Korea).

### 2.5. Immunoblot Analysis

After washing with cold sterile PBS, cells were lysed with lysis buffer containing 20 mM Tris-Cl (pH 7.5), 1% Triton X-100, 137 mM sodium chloride, 1 mM sodium orthovanadate, 10% glycerol, 2 mM EDTA, 25 mM *β*-glycerolphosphate, 1 mM phenylmethylsulfonylfluoride, 2 mM sodium inorganic pyrophosphate, and 1 *μ*g/mL leupeptin. Total cell lysates were separated using SDS polyacrylamide gel and electrophoretically transferred to nitrocellulose membranes. After serial incubation of the membranes with primary antibodies and horseradish peroxidase- (HRP-) conjugated anti-IgG antibodies, the membranes were exposed to enhanced chemiluminescence (ECL) reagent (Millipore, Burlington, MA, USA), and images were obtained using LAS3000 mini (Fujifilm, Tokyo, Japan).

### 2.6. 2-Deoxyglucose- (2-DG-) Uptake Assay

Cells seeded in a 96-well plate (2 × 10^4^ cells/well) were incubated with 1 mM 2-DG for 10 min. 2-deoxyglucose-6-phosphate (2-DGP) was determined using Glucose uptake-Glo assay kit (Promega, Madison, WI, USA) following the manufacturer's instruction.

### 2.7. Determination of NAD^+^/NADH Ratio

Cells were plated in a 96-well plate and treated with the indicated concentration of phenformin for 24 h. Using NAD^+^/NADH Glo Assay (Promega), the NAD^+^/NADH ratio was calculated [6].

### 2.8. Determination of Glucose Consumption in Culture Medium

Cell culture media were collected 24 h after incubation, and a 1000-fold diluted solution was achieved by adding ice-cold 50% methanol. The solution was introduced to 1 equivalent volume of chloroform for phase separation. Samples were vortexed for 10 s and subsequently centrifuged at 16,000 rcf for 5 min. The upper hydrophilic phase was transferred to a microcentrifuge tube, and the extraction was repeated once with the remaining lower phase. The collected hydrophilic phase was dried under gentle stream of nitrogen. Dried samples were reconstituted with 100 *μ*L methoxyamine hydrochloride (20 mg/mL, Sigma Aldrich) in pyridine and incubated at 37°C for 90 min. Following cool-down, samples were introduced to 100 *μ*L N,O-bis(trimethylsilyl)trifluoroacetamide solution with 1% trimethylchlorosilane (Sigma Aldrich) and derivatized at 60°C for 40 min. Glucose contents were analyzed by the Shimadzu GCMS-QP2010 (Tokyo, Japan) system equipped with DB-5MS (30 m, 0.25 mm, 0.25 *μ*m; Agilent Technologies, DE, USA). Inlet temperature was set at 270°C, and the samples were injected in split mode (1 : 2). Column oven temperature was maintained as the following gradient: 0 min, 80°C; 2 min, 80°C; 7 min, 100°C; 10 min, 100°C; 35 min, 200°C; 36 min, 200°C; 48.5 min, 300°C; and 50.5 min, 300°C. Mass scan ranged from 40 to 600 m/z with 3.06 scan/s of scan rate. Absolute quantitative values were calculated with peak area based on calibration curve by external glucose standard solutions and normalized by total protein contents.

### 2.9. Determination of Intracellular Lactate and Aspartate Levels

The sample injection volume was 5 *μ*L, and peak separation was performed on a Hypersil GOLD C_8_ (2.1 × 150 mm, 5 *μ*m, Thermo Scientific, Waltham, MA) maintained at 30°C. The analysis was performed to gradient condition using Agilent 1290 infinity II system with autosampler, column oven, and binary pump (HPLC water containing 0.01% (*v*/*v*) formic acid, A; 100% methanol, B), and flow rate was 0.2 mL/min. LC-MS/MS data were acquired with an Applied Biosystems SCIEX 4000 QTRAP hybrid triple quadrupole-linear ion trap mass spectrometer equipped with a Turbo V ionization source. The detection was conducted using multiple reaction monitoring (MRM) of the transitions of m/z 89 > 43 for lactate, m/z 132 > 88 for aspartate, and m/z 157 > 112 for ^13^C_5_, D_5_, ^15^N-glutamate (ISTD) in the negative ion mode. Acquisition and analysis data were performed with Analyst® software (ver.1.6.2; Applied Biosystems, Foster City, CA, USA).

### 2.10. Determination of Mitochondria ATP Production Rate, Oxygen Consumption Rate (OCR), and Extracellular Acidification Rate (ECAR)

OCR and ECAR were determined using XFp analyzer (Seahorse Bioscience, North Billerica, MA, USA). XFp cell mito-stress test kit, XFp glycolysis stress test kit, and XFp Real-Time ATP rate assay kit (Seahorse Bioscience) were used to detect OCR, ECAR, and ATP ratio, respectively. All the reagents and assay conditions were followed by manufacturer's instructions.

### 2.11. RNA Preparation and RNA Sequencing

Total RNA was extracted from HCC827 and HCC827 GR cells using TRIzol reagent (Invitrogen). Purity and concentration of RNA samples were evaluated by NanoDrop Lite (Thermo Scientific, MA, USA), and transcriptome RNA-sequencing of the samples was performed by Macrogen.

### 2.12. Transmission Electron Microscopy

Cells were fixed with Karnovsky's fixative and fixed with 2% osmium tetroxide. 0.5% uranyl acetate was used for staining, and propylene oxide and ethanol were used for dehydration. By using Spurr's resin, cells were embedded and polymerized at 70°C. After embedding, blocks were trimmed with ultramicrotome (EM UC7, Leica, Wetzler, Germany) and detected with Transmission Electron Microscope (JEM1010, JEOL, Tokyo, Japan).

### 2.13. Flow Cytometry Analysis to Determine Mitochondria Membrane Potential

Tetramethylrhodamine methyl ester perchlorate (TMRM) (Sigma-Aldrich, MO, USA) was prepared as 100 *μ*M stock solution in dimethyl sulphoxide. Cancer cells were incubated with 100 nM TMRM for 30 min at 37°C. After trypsin treatment, the detached stained cells were analyzed by Novocyte flow cytometer (Agilent, CA, USA).

### 2.14. Xenograft Assay

Five-week-old male Balb/c-nu mice were supplied from Raon Bio Inc. (Seoul, South Korea). Animal studies were performed according to the institute regulation and approval from Seoul National University Institutional Animal Care and Use Committee (Approval #: SNU-170717-6-1). Mice were kept in SNU semipathogen-free animal facility, with five mice in each cage. After anesthesia with Zoletil®/Rompun® mixture, 3.5 × 10^6^ HCC827 or 3 × 10^6^ HCC827 GR cells were inoculated on right flanks of mice. One week after inoculation, the mice were randomly divided into two groups (vehicle control group and phenformin treatment group (300 mg/kg/day, P.O.)). Animal number in xenograft study is 6-10 per group. Mice were monitored every other day, tumor length and width were detected by calipers, and tumor volume was calculated using the formula (length × width^2^) × 0.5. Animals were sacrificed by carbon dioxide inhalation in euthanasia chamber.

### 2.15. Statistics

Student's *t*-test was performed to compare the difference between two groups. One-way ANOVA and Tukey's post hoc was used to analyze differences in multiple comparison. Statistical analysis was calculated using SigmaPlot (version 12.5). The statistical significance was accepted at ∗*P* < 0.05, ∗∗*P* < 0.01, and ∗∗∗*P* < 0.001; ^#^*P* < 0.05, ^##^*P* < 0.01, and ^###^*P* < 0.001.

## 3. Results

### 3.1. Long-Term Treatment with EGFR-TKIs Induces Acquired Resistance with Downregulation of EGFR Signaling in Lung Cancer Cells

**Figure 1:**
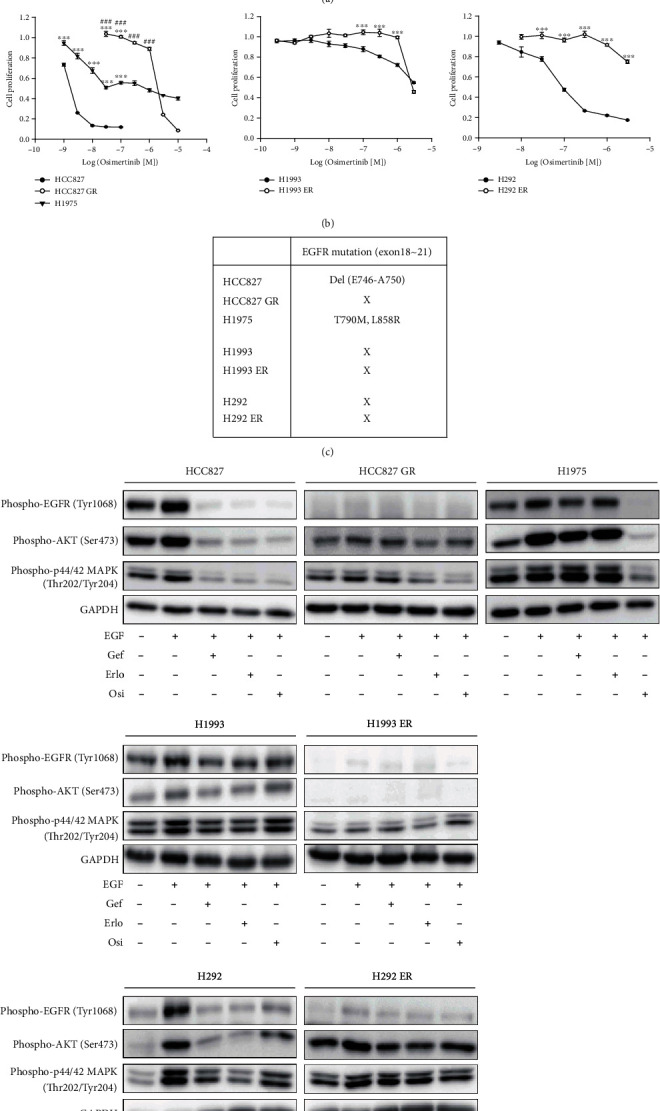
Acquisition of EGFR-TKI resistance by long-term treatment of gefitinib or erlotinib in NSCLCs. (a and b) Effects of EGFR TKIs on cell proliferation of EGFR TKI-resistant lung cancer cells. HCC827, HCC827 GR, H1993, H1993 ER, H292, H292 ER, and H1975 cells were incubated with various concentrations of gefitinib or erlotinib (a) and osimertinib (b), and cell proliferation was monitored for 72 h by IncuCyte ZOOM analyses. Data represent means ± S.E.M. (*n* = 3 − 6, ∗*P* < 0.05, ∗∗*P* < 0.01, ∗∗∗*P* < 0.001 vs. parental cell line; ^###^*P* < 0.001 vs. H1975). (c) Genomic DNA sequencing of EGFR exon 18 to 21. (d) EGFR and its downstream signaling activities in HCC827, HCC827 GR, H1993, H1993 ER, H292, and H292 ER cells. All cells were pretreated with vehicle or 100 nM gefitinib, erlotinib, or osimertinib for 1 h and then exposed to 100 ng/mL EGF for 5 min. Total cell lysates were subjected to immunoblottings for phospho-EGFR (Tyr1068), phosphor-AKT (Ser473), or phosphor-p44/p42 MAPK (Thr202/Tyr204).

Gefitinib-resistant NSCLC HCC827 (HCC827 GR) cells were established via long-term exposure of the cells to stepwise escalation of gefitinib. HCC827 GR cells exhibited resistance to gefitinib at a level similar to that in H1975 cells (T790M mutation). Other lung cancer cell lines, H1993 (NSCLC) and H292 (mucoepidermoid lung cancer) cells, also exhibited resistance to erlotinib after long-term treatment (H1993 ER and H292 ER, respectively) (Figure 1(a)). Osimertinib, a third-generation EGFR-TKI, strongly inhibited H1975 cell proliferation but marginally affected cell proliferation in the lung cancer cell lines with acquired EGFR-TKI resistance (HCC827 GR, H1993 ER, and H292 ER) (Figure 1(b)). Because various mutations in the EGFR tyrosine kinase domain associated with gefitinib resistance have been reported [7], DNA sequencing of EGFR exons 18 to 21 was performed for HCC827, HCC827 GR, H1975, H1993, H1993 ER, H292, and H292 ER cells. An exon 19 deletion mutation and inherited gefitinib resistance, T790M, were found in HCC827 and H1975 cells, respectively (Figure 1(c)). However, mutations were not detected in all the lung cancer cells with acquired EGFR-TKI resistance tested (Figure 1(c)). Furthermore, EGF-induced phosphorylation of EGFR (Tyr1068) and its downstream kinases, phosphorylation of AKT (Ser473) or p44/p42 MAPK (Thr202/Tyr204), was not observed or was weaker in the three acquired-resistance cells compared with their parental cell lines. In addition, the EGFR tyrosine kinase-inhibiting effects of gefitinib, erlotinib, and osimertinib were weaker (Figure 1(d)). Conversely, EGFR phosphorylation and its downstream signals were successfully suppressed by osimertinib but not by gefitinib or erlotinib in H1975 cells (T790M) (Figure 1(d)). The results showed that the acquisition of EGFR-TKI resistance by long-term treatment with gefitinib or erlotinib led to relatively minimal effects on EGFR activity, and these cells subsequently exhibited decreased sensitivity to EGFR-TKIs, even osimertinib.

### 3.2. Glycolysis Activity Is Decreased in Lung Cancer Cells with Acquired EGFR-TKI Resistance

**Figure 2:**
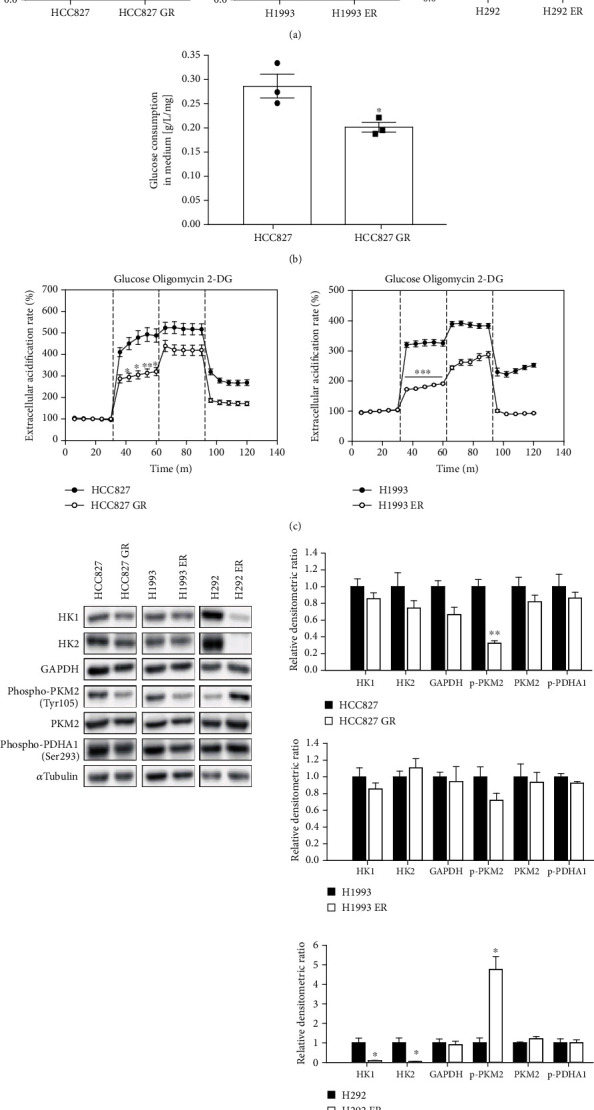
Decreased glycolysis activity in acquired EGFR-TKI-resistant lung cancer. (a) Relative glucose consumption by 2-DG uptake assay. Data represent means ± S.E.M. (*n* = 3, ∗∗*P* < 0.01, ∗∗∗*P* < 0.001 vs. 2-DG uptake in parental cell line). (b) Glucose contents in culture media. Glucose level was determined by GC-MS in culture media from HCC827 and HCC827 GR cells incubated for 24 h. Glucose concentration was normalized by total protein amounts. Data represent means ± S.E.M. (*n* = 3, ∗*P* < 0.05 vs. HCC827). (c) ECAR changes. ECAR values were obtained from glycolysis stress test using XFp analyzer. Glucose (10 mM), oligomycin (1 *μ*M), and 2-DG (50 mM) were added at indicated time points. Data represent means ± S.E.M. (*n* = 3, ∗*P* < 0.05, ∗∗*P* < 0.01, ∗∗∗*P* < 0.001 vs. parental cell line). (d) Basal protein expression levels of glycolysis enzymes. Protein expression of hexokinase (HK)1, HK2, GAPDH, phosphor-PKM2 (Tyr105), PKM2, and phospho-PDHA1 (Ser293) was assessed by immunoblottings.

EGFR signaling promotes aerobic glycolysis in triple-negative breast cancer (TNBC) [8], and the inhibition of EGFR reverses the Warburg effect and reactivates oxidative phosphorylation (OXPHOS) in NSCLC cells [9]. Because EGFR signaling is suppressed in the three acquired-resistance lung cancer cell lines (HCC827 GR, H1993 ER, and H292 ER), we examined glucose metabolism in these cancer cells. The 2-DG uptake assay showed that glucose uptake in all three resistant cancer cell lines was decreased (Figure 2(a)), and the glucose consumption assay showed that the amount of glucose remaining in the culture medium was higher in HCC827 GR cells compared with HCC827 parental cells (Figure 2(b)). Using the XFp analyzer with a glycolysis stress kit, changes in the extracellular acidification rate (ECAR) representing glycolysis were estimated. After being exposed to the same amount of glucose (10 mM), HCC827 GR and H1993 ER cells showed less changes compared with their parental cells (Figure 2(c)). Furthermore, expression levels of enzymes involved in glycolysis (HK1, HK2, and GAPDH) were lower in the two resistant cell lines (HCC827 GR and H1993 ER) (Figure 2(d)), whereas the dimer (inactive) form of Tyr105-phosphorylated PKM2 was slightly reduced in HCC827 GR and H1993 ER cells (Figure 2(d)). Notably, H292 ER cells lacked both HK1 and HK2, first-step enzymes in glycolysis, and the Tyr105-phosphorylated PKM2 level was upregulated (Figure 2(d)), indicating that glycolysis dysfunction in the H292 ER cells was mainly due to HK1/2 defects. The mRNA expression associated with the glycolysis gene set was further analyzed using RNA sequencing for both HCC827 and HCC827 GR cells. However, mRNA expression patterns associated with glycolysis in the two cell types did not exhibit any specific trends (Figure S1A). Although metabolic differences were not clearly identified at the mRNA or protein level in glycolytic enzymes, the metabolic data indicate that glycolysis capacity was decreased in the lung cancer cells with acquired EGFR-TKI resistance.

### 3.3. Reactivation of Mitochondrial OXPHOS Function in Lung Cancer Cells with Acquired EGFR-TKI Resistance

**Figure 3:**
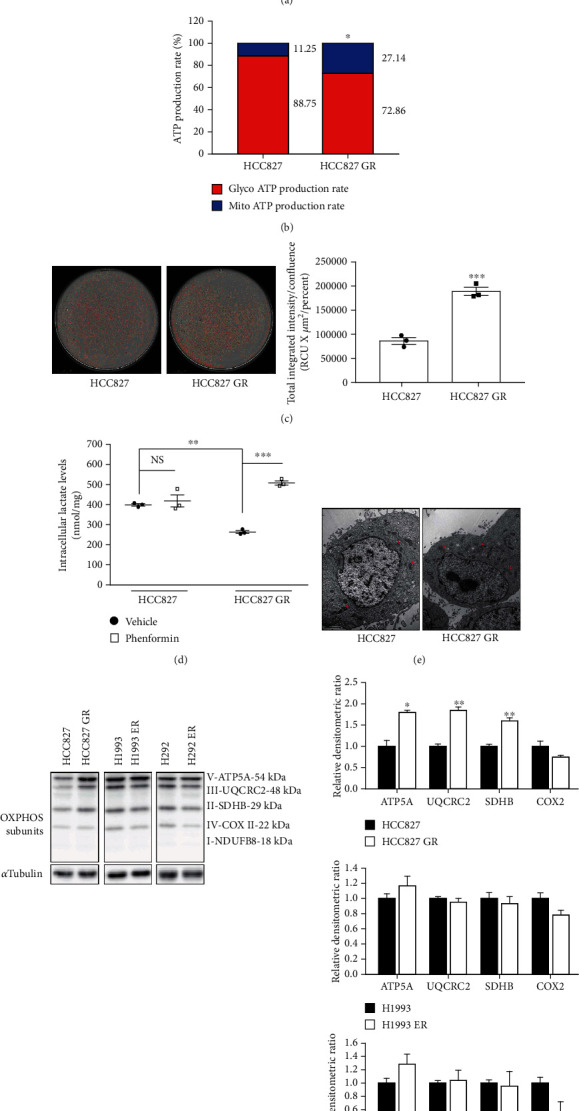
Reactivation of mitochondria function in acquired EGFR-TKI-resistant lung cancer. (a) OCR values were measured by XFp analyzer with cell mito stress kit. Oligomycin (1.5 *μ*M), FCCP (0.5 *μ*M), and mixture of rotenone (Ro, 0.5 *μ*M) and antimycin A (AA, 0.5 *μ*M) were treated at indicated time points. Data represent means ± S.E.M. (*n* = 3, ∗∗∗*P* < 0.001 vs. parental cell line). (b) Relative contribution of glycolysis and OXPHOS to ATP production. Using XFp real-time ATP rate assay kit, ATP production rate from glycolysis and OXPHOS was simultaneously determined in HCC827 and HCC827 GR cells. Data represent means (*n* = 3, ∗*P* < 0.05 vs. mito ATP production rate in HCC827). (c) Mitochondrial membrane potential. HCC827 and HCC827 GR cells were incubated with 100 nM TMRM for 30 min, and fluorescence signals were detected by IncuCyte ZOOM. Total integrated intensity of TMRM (red fluorescence) was normalized with cell confluence (outlined with yellow line). Data represent means ± S.E.M. (*n* = 3, ∗∗∗*P* < 0.001 vs. HCC827). (d) Intracellular lactate levels. HCC827 and HCC827 GR cells were treated with 100 *μ*M phenformin for 24 h, and lactate levels in cell lysates were determined by LC-MS/MS. Intracellular lactate level was normalized with total protein amounts. Data represent means ± S.E.M. (*n* = 3, ∗∗*P* < 0.01, ∗∗∗*P* < 0.001 significant difference between the two indicated groups). (e) Number and size of mitochondria (red arrows) in HCC827 and HCC827 GR cells were analyzed by TEM. (f) Protein level of OXPHOS subunits (ATP5A, UQCRC2, SDHB, COX II, and NDUFB8) was detected by immunoblotting.

Because glycolysis is the main energy process using glucose in rapidly growing cancers, glycolysis is considered a hallmark of cancer (Warburg effect) [10]. However, the role of mitochondrial OXPHOS in cancer progression has also been studied [4, 5, 11, 12]. Because EGFR inhibition induces the reactivation of mitochondrial OXPHOS in NSCLC cells [9], and the lung cancer cells with acquired EGFR-TKI resistance exhibited decreased EGFR kinase signaling activities (Figure 1(d)), OXPHOS activities in the three lung cancer cell lines with acquired EGFR-TKI resistance were compared with those in their parental cell lines. Using the XFp analyzer with a cell mito stress test kit, oxygen consumption rate (OCR) changes were monitored after treatment with OXPHOS modulators (1.5 *μ*M oligomycin, 0.5 *μ*M trifluoromethoxy carbonylcyanide phenylhydrazone (FCCP), and a mixture of 0.5 *μ*M rotenone and 0.5 *μ*M antimycin A). ATP-linked mitochondrial respiration increased in HCC827 GR and H1993 ER cells compared with their parental cells (Figure 3(a)). In H292 ER cells, maximal respiration in mitochondria increased but ATP-associated respiration did not change significantly compared with the parental cells (Figure 3(a)). Then, the ATP production ratio was compared between glycolysis and OXPHOS using the XFp analyzer with an ATP real-time rate assay kit. ATP production in the HCC827 cells was highly dependent on glycolysis (88.75%), but not on OXPHOS (11.25%). However, HCC827 GR cells had more ATP production that depended on mitochondrial OXPHOS (27.14%) than did HCC827 cells (Figure 3(b)). Mitochondrial membrane potential is induced by a proton pump in OXPHOS and regarded as an essential component during mitochondrial ATP production. Mitochondrial membrane potential in HCC827 and HCC827 GR cells was analyzed via staining with tetramethylrhodamine methyl ester (TMRM, red dots). As shown in Figure 3(c), total integrated red fluorescence intensity versus cell confluence was significantly higher in HCC827 GR cells. Conversely, the basal intracellular level of lactate, the end product of glycolysis, was significantly decreased in HCC827 GR cells compared with HCC827 cells (Figure 3(d)). Inhibition of OXPHOS can cause a compensative increase in glycolysis resulting in the conversion of pyruvate to lactate. The lactate level increased in phenformin- (OXPHOS inhibitor-) treated HCC827 GR cells; however, a difference in HCC827 cells was not detected (Figure 3(d)). These results indicate that mitochondrial OXPHOS was reactivated in the NSCLC cell lines with acquired EGFR-TKI resistance (HCC827 GR and H1993 ER). Next, we assessed morphological changes in the mitochondria as well as the protein and mRNA expression of OXPHOS subunits. Transmission electron microscopy images showed no differences in the size and number of mitochondria in both HCC827 and HCC827 GR cells (Figure 3(e)). The expression of OXPHOS subunit proteins (complex 2, 3, 5) was slightly increased in HCC827 GR cells compared with HCC827 cells; however, the OXPHOS subunits expression in H1993 ER and H292 ER cells was not significantly different from that in their parental cells (Figure 3(f)). The distribution of expression levels of different mRNAs associated with OXPHOS subunits was viewed as a MA plot. Significant fold-changes between HCC827 and HCC827 GR cells were not found (Figure S1B). The data indicated that increased OXPHOS capacity in EGFR-TKI-resistant NSCLC is associated with enhanced mitochondrial function but not with changes in related gene expression.

### 3.4. Inhibition of Proliferation in Lung Cancer Cells with Acquired EGFR-TKI Resistance Is Caused by Phenformin

**Figure 4:**
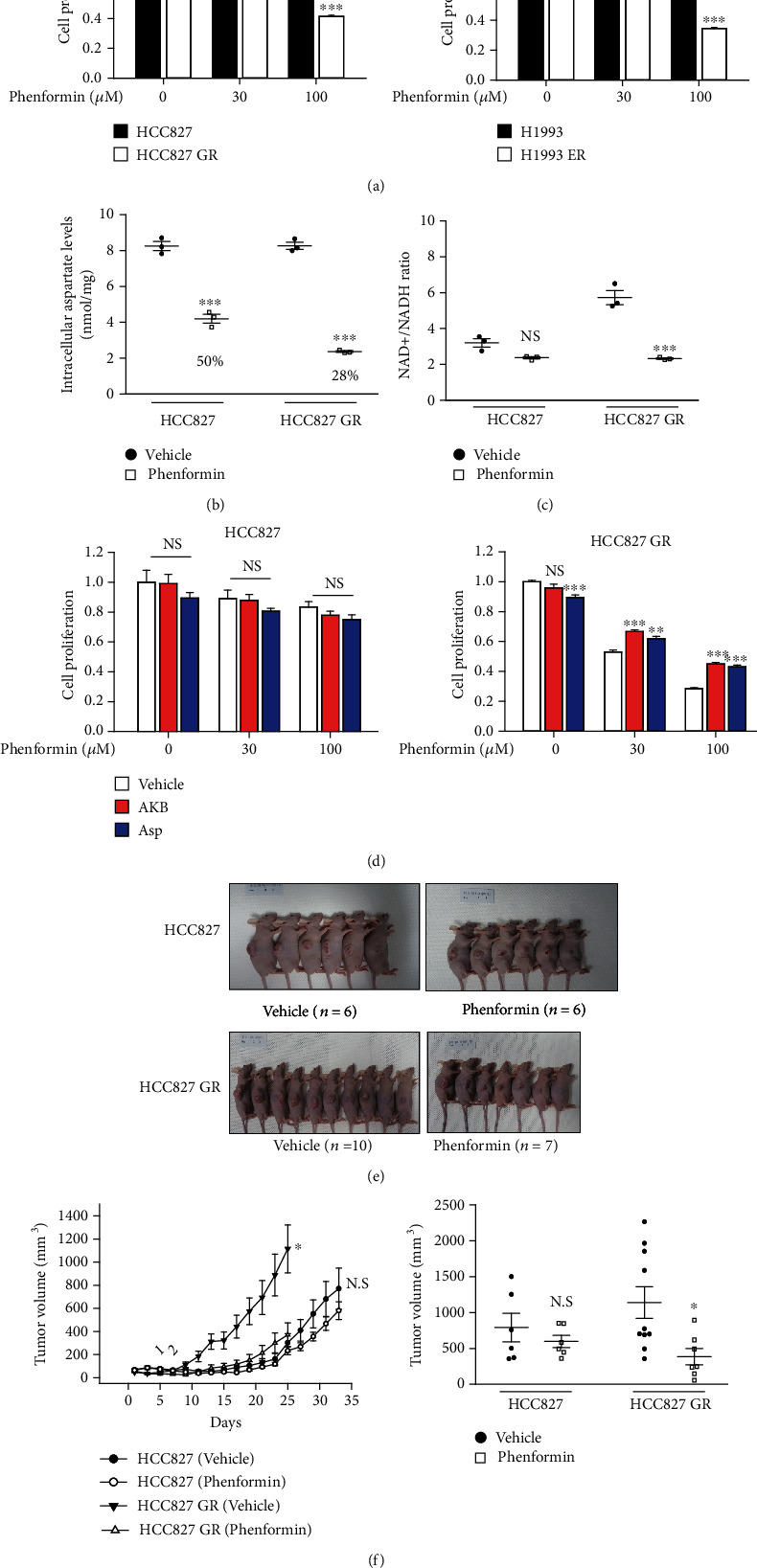
Selective anticancer effect of phenformin in acquired EGFR-TKI-resistant lung cancer. (a) HCC827, HCC827 GR, H1993, and H1993 ER cells were exposed to phenformin (30 and 100 *μ*M) for 72 h, and cell proliferation was monitored by IncuCyte ZOOM. Data represent means ± S.E.M. (*n* = 6, ∗∗∗*P* < 0.001 vs. parental cell line). (b) Intracellular aspartate level. HCC827 and HCC827 GR cells were treated with 100 *μ*M phenformin for 24 h, and aspartate levels in cell lysates were determined by LC-MS/MS. Data represent means ± S.E.M. (*n* = 3, ∗∗∗*P* < 0.001 vs. vehicle-treated group). (c) Potentiation of redox stress by phenformin in acquired EGFR-TKI-resistant cancer cells. Intracellular NAD^+^/NADH ratio was analyzed in HCC827 and HCC827 GR cells 24 h after exposure with vehicle or 100 *μ*M phenformin. Data represent means ± S.E.M. (*n* = 3, ∗∗∗*P* < 0.001 vs. vehicle-treated group). (d) Reversal of antiproliferative effect of phenformin by electron acceptor or aspartate. Cell proliferation was monitored for 72 h in HCC827 and HCC827 GR cells treated with phenformin (30 and 100 *μ*M) in the presence or absence of 1 mM AKB or 10 mM aspartate. Data represent means ± S.E.M. (*n* = 6, ∗∗*P* < 0.01, ∗∗∗*P* < 0.001 vs. vehicle-treated group). (e and f) *In vivo* anticancer effect of phenformin on tumor growth of EGFR-TKI-resistant lung cancer. HCC827 and HCC827 GR cells were inoculated into right flank of Balb/c nude mice, and the mice were orally administered with 300 mg/kg phenformin or tap water (vehicle) once a day. (e) Representative images. (f) Tumor volumes were measured every other day. Data represent means ± S.E.M. (*n* = 6 − 10, ∗*P* < 0.05 vs. vehicle-treated group).

Biguanides, the most prescribed anti-diabetic agents, have been recognized for their anticancer effects, and many clinical trials are currently in progress [13–16]. The repositioning of biguanides as anticancer agents is attracting much attention due to the cost benefit and minimal safety issues. In a recent study, the combination of osimertinib with phenformin delayed osimertinib resistance in a preclinical NSCLC model [17]. We hypothesized that phenformin, an OXPHOS complex 1 inhibitor, would selectively inhibit the proliferation of lung cancer cells with acquired EGFR-TKI resistance that mainly employ mitochondrial OXPHOS. A prototype biguanide, phenformin, more effectively inhibited the proliferation of HCC827 GR and H1993 ER cells than their parental cell lines (Figure 4(a)). However, the growth-inhibiting effect of phenformin was not enhanced in H292 ER cells compared with H292 cells (Figure S2C), which is consistent with the data showing no difference in ATP-associated OCR change (Figure 3(a)). Next, the potential mechanism by which phenformin inhibits the proliferation of EGFR-TKI-resistant NSCLC cells was assessed. Because OXPHOS is required for aspartate biosynthesis in proliferating cells [6, 18], intracellular aspartate level was measured after exposing HCC827 and HCC827 GR cells to phenformin. The intracellular aspartate level decreased to a greater extent in HCC827 GR cells than in HCC827 cells after exposure to phenformin (Figure 4(b)). Inhibition of aspartate biosynthesis occurs due to an imbalance in the NAD^+^/NADH ratio. When the cellular NAD^+^/NADH ratio was measured, phenformin-mediated NAD^+^/NADH imbalance was only observed in HCC827 GR cells (Figure 4(c)). *α*-Ketobutyrate (AKB) is a representative electron acceptor that participates in regenerating NAD^+^ [6]. The addition of both AKB and aspartate partially alleviated the growth-inhibiting effects of phenformin in HCC827 GR cells (Figure 4(d)). To assess if mitochondrial OXPHOS complex I is a target of phenformin, the effects of rotenone, a potent OXPHOS complex 1 inhibitor, were investigated. TMRM-based mitochondria membrane potential in HCC827 GR cells was more potently reduced by rotenone treatment than HCC827 cells (Figure S2A). As expected, rotenone exerted strong growth-inhibiting effects in HCC827 GR cells, and its antiproliferative effect was reversed with AKB treatment (Figure S2B). To evaluate the anticancer effects of phenformin *in vivo*, Balb/c nude mice were implanted with HCC827 or HCC827 GR cells. In xenografts inoculated with HCC827 GR cells, oral administration of phenformin (300 mg/kg/day) significantly reduced tumor growth derived from HCC827 GR cells. Conversely, phenformin administration did not significantly affect tumor growth in xenografts inoculated with HCC827 cells (Figures 4(e) and 4(f)).

### 3.5. Reversal of Anticancer Effects of Phenformin Is Caused by Glycolysis Reactivation in Lung Cancer Cells with Acquired EGFR-TKI Resistance

**Figure 5:**
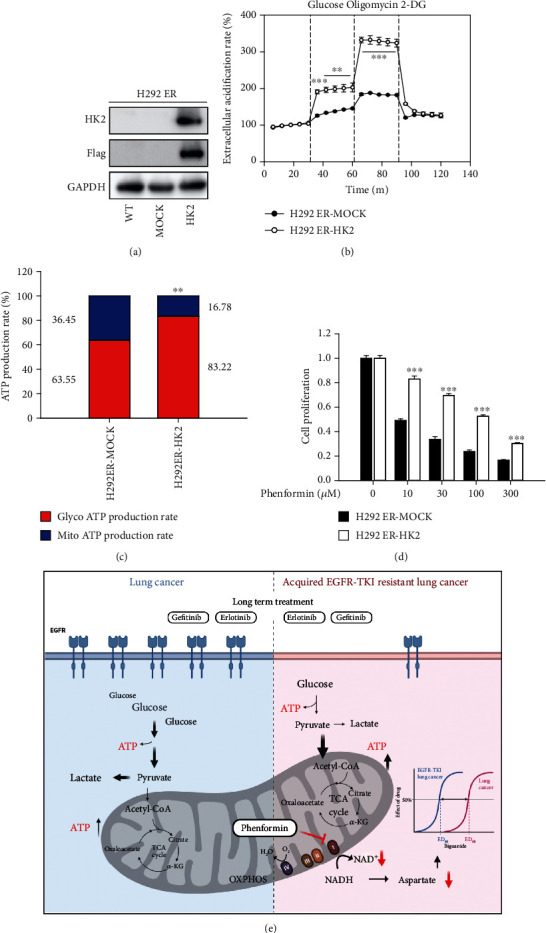
Reversal of antiproliferation effect of phenformin by glycolysis reactivation. (a) Establishment of hexokinase2 overexpressing H292 ER cells (H292 ER-HK2). H292 ER cells were transfected with pCAG-Flag-HK2-IRES-Blas or pCAG-Flag-IRES-Blas and protein expression was confirmed by immunoblotting. (b) ECAR values in H292 ER-MOCK and H292 ER-HK2 cells. Glycolysis stress test was performed by XFp analyzer, and ECAR was measured and normalized with basal ECAR level. Data represent means ± S.E.M. (*n* = 3, ∗∗*P* < 0.01, ∗∗∗*P* < 0.001 vs. H292 ER-MOCK). (c) Relative contribution of glycolysis and OXPHOS to ATP production in H292 ER-MOCK and H292 ER-HK2 cells. Using XFp real-time ATP rate assay kit, ATP production rate from glycolysis and OXPHOS was measured in H292 ER-MOCK and H292 ER-HK2 cells. Data represent means (*n* = 3, ∗∗*P* < 0.01 vs. mito ATP production rate in H292 ER-MOCK). (d) Alleviated antiproliferative effect of phenformin by HK2 overexpression. H292 ER-MOCK and H292 ER-HK2 cells were treated with vehicle or phenformin and cell proliferation rate was monitored for 72 h by IncuCyte ZOOM. Data represent means ± S.E.M. (*n* = 6, ∗∗∗*P* < 0.001 vs. H292 ER-MOCK). (e) Schematic illustration for metabolic shift to OXPHOS and the enhanced biguanide responsiveness in acquired EGFR-TKI resistant lung cancer.

Because the reactivation of OXPHOS in most of the lung cancer cell lines with acquired EGFR-TKI resistance was observed, we hypothesized that phenformin sensitivity could be diminished by the restoration of glycolysis (Warburg effect) in the resistant cell types. Hexokinase (HK) is a first-step glycolytic enzyme that converts glucose into glucose 6-phosphate. Among the five HK isoforms, HK2 is highly expressed and functions as the predominant form in cancer cells [19]. Because HK2 and HK1 expressions were absent in H292 ER cells (Figure 2(d)), we hypothesized that HK enzyme deficiency is a key event for the metabolic shift to OXPHOS in H292 ER cells. HK2-overexpressing H292 ER cells (H292 ER-HK2) were established by transfection with a Flag-tagged human HK2 overexpression vector (Figure 5(a)). XFp analysis with a glycolysis stress test kit showed that ECAR changes representing glycolysis and its capacity were greater in H292 ER-HK2 cells compared with mock-transfected cells (Figure 5(b)). The ATP production ratio from glycolysis also increased from 63.55% to 83.22% (Figure 5(c)). Furthermore, the inhibitory effects of phenformin on the proliferation of H292 ER cells were significantly decreased by HK2 overexpression, indicating that enhanced glycolysis reduces the anticancer effects of phenformin in lung cancer cells with acquired EGFR-TKI resistance (Figure 5(d)).

## 4. Discussion

EGFR overexpression has been detected in approximately half of NSCLC patients and is associated with poor prognosis, whereas EGFR-TKIs such as gefitinib and erlotinib significantly increase the survival rate of patients without serious side effects (6). Unfortunately, most patients acquire resistance to EGFR-TKIs within 6–12 months [20, 21]. Several heterogeneous mechanisms, such as *de novo* mutation, amplification, and downstream pathway activation of EGFR or the activation of bypass pathways (e.g., pathways involving c-Met, Axl, insulin-like growth factor receptor, and other members of the EGFR family), explain the acquired EGFR-TKI resistance in NSCLC [22–25]. Osimertinib (Tagrisso®, a third-generation EGFR-TKI) has been approved for the treatment of patients with metastatic and EGFR T790M mutation-positive NSCLC [26]. However, several cases of patients resistant to osimertinib have been reported in clinics [26]. Although phenotype characteristics of EGFR-TKI-resistant lung cancer cells can be classified as EGFR-dependent and EGFR-independent growth [26], deciding an appropriate treatment strategy is difficult due to the acquired resistance being driven by heterogeneous mechanisms. Herein, we propose a plausible therapeutic strategy targeting the cancer metabolism of EGFR-TKI-resistant lung cancer cells that focuses on the metabolic shift to mitochondrial OXPHOS.

In the tested lung cancer cell lines, the metabolic use of glucose differed between EGFR-TKI-sensitive and EGFR-TKI-resistant lung cancer cells. Both glucose uptake and glycolytic capacity were significantly reduced in lung cancer cell lines with acquired EGFR-TKI resistance compared with their parental cell lines. Conversely, the overall function of mitochondria was enhanced in the resistant cell lines, as evidenced by increases in mitochondrial ATP production and mitochondrial membrane potential. Reportedly, EGFR stimulation accelerates glycolysis in TNBC cell lines [8]. Furthermore, suppression of EGFR signaling in NSCLC cells reverses the Warburg effect and reactivates OXPHOS [9]. Because EGFR-TKI-resistant lung cancer cell lines (HCC827 GR, H1993 ER, and H292 ER) acquired resistance via stepwise exposure to EGFR-TKIs over a long-term period [21, 27] and exhibited downregulated signaling activity of EGFR (Figure 1(d)), compensatory activation of mitochondrial OXPHOS in the resistant NSCLC cell lines appears reasonable.

Cancer metabolism is an emerging research area in cancer biology. Based on the reprogrammed pathways of nutrient utilization and metabolism in cancer cells, several metabolic characteristics have been identified as hallmarks of cancer and recognized as promising therapeutic targets for cancer chemotherapy [10, 28–32]. The metabolic processes of mitochondria have attracted attention as part of the development of treatment strategies targeting cancer metabolism [5]. In particular, biguanides inhibiting mitochondrial complex I could be of interest because metformin has been used for an extended period as an effective type 2 diabetes agent, and its safety is proven [14]. However, clear evidence showing the successful anticancer efficacy of biguanides has not been reported in numerous clinical trials, which may be due to the nonselection of either a proper biomarker that determines the reactivity of biguanide or an appropriate biguanide-responding carcinoma [33–35]. In the present study, phenformin strongly inhibited cell proliferation and tumor growth in NSCLC with acquired EGFR-TKI resistance (Figure 4).

We demonstrated that the anticancer effects of phenformin depend on the metabolic status of cancer cells, ATP production via glycolysis, or mitochondrial OXPHOS (Figure 5(c)). Although diverse cellular proteins including AMP-activated protein kinase, liver kinase B1 [36], OXPHOS complex 1 [37], and mitochondrial glycerolphosphate dehydrogenase [38] have been suggested as pharmacological targets of biguanides, we considered the NAD^+^/NADH imbalance caused by OXPHOS complex 1 inhibition a plausible mechanism by which phenformin acts on EGFR-TKI-resistant NSCLC. Because NAD^+^ is required to activate cytosolic malate dehydrogenase to generate oxaloacetate and then for the synthesis of aspartate through aspartate aminotransferase, a key function of mitochondrial OXPHOS and the redox balance in cancer cells is the biosynthesis of aspartic acid for the rapid growth of cancer [6, 18]. As shown in Figure 4, the intracellular aspartate level was decreased by phenformin in EGFR-TKI-resistant NSCLC. Because the metabolic shift to mitochondrial OXPHOS is triggered by long-term exposure to EGFR-TKIs, biguanide targeting OXPHOS may result in sustained redox stress as well as a subsequent aspartic acid deficiency and can be proposed as a new therapeutic option for NSCLC with acquired EGFR-TKI resistance. Consistent with our data, the combination of phenformin with osimertinib delayed the occurrence of resistance in a preclinical model of NSCLC in a recent study [17].

The present study has several limitations. First, all lung cancer cell lines did not exhibit a metabolic shift from glycolysis to mitochondrial OXPHOS under long-term exposure to EGFR-TKIs. In fact, phenformin sensitivity in H292 ER cells was of a similar intensity to that of its parental H292 cells (Figure S2C). Second, a proper biomarker indicating if a metabolic shift had occurred was not suggested. The expression levels of glycolytic enzymes, such as HK1, HK2, and GAPDH, were downregulated in HCC827 GR and H1993 ER cells. Conversely, Tyr105-phosphorylated PKM2, an inhibitory form of PKM2, and S293-phosphorylated PDHA1, which converts pyruvate into acetyl-CoA, were also downregulated in HCC827 GR and H1993 ER cells. Based on our results showing that the expression levels of glycolytic enzymes, such as HK1 and HK2, were low and those of the inactive form of PKM2 (Tyr105-phosphorylated PKM2) and S293-phosphorylated PDHA1 were high, H292 ER cells should be more dependent on OXPHOS and more vulnerable to phenformin. However, significant differences were not observed in terms of inhibitory effects of phenformin on the proliferation of H292 and H292 ER cells (Figure S2C). Because the metabolic processes of cancer cells are diverse, elucidating an indicator that defines the metabolic characteristics of cancer is challenging. Although specific genes that uniquely control the metabolic status of cancer cells have been successfully elucidated in a few studies [33, 35, 39], we could not find a common metabolic point for which mitochondrial function is upregulated in three different EGFR-TKI-resistant lung cancer cell lines. However, we presume that biguanide sensitivity in NSCLC with acquired EGFR-TKI resistance relies on mitochondrial OXPHOS activity. Because mitochondrial function is precisely controlled by numerous enzymes involved in several biochemical cycles, predicting the responsiveness to biguanide by only assessing specific gene expression is difficult.

## 5. Conclusions

Long-term treatment with EGFR-TKIs induces chemoresistance with a metabolic shift from glycolysis to OXPHOS in lung cancer cells. Suppressing OXPHOS by phenformin causes redox imbalance, leading to inhibition of aspartate biosynthesis and ultimately cancer cell growth. Our research provides pharmacological evidence for a therapeutic strategy using biguanides for EGFR-TKI-resistant NSCLC (Figure 5(e)).

## Data Availability

Data is contained within the article or supplementary material.

## Abbreviations

2-DG: 2-Deoxyglucose
AA: Antimycin A
AKB: Sodium 2-oxobutyrate
Asp: Aspartate
ECAR: Extracellular acidification rate
EGF: Epidermal growth factor
EGFR: Epidermal growth factor receptor
Erlo: Erlotinib
FCCP: Trifluoromethoxy carbonylcyanide phenylhydrazone
GAPDH: Glyceraldehyde-3-phosphate dehydrogenase
Gef: Gefitinib
H1993 ER: Erlotinib-resistant H1993 cells
H292 ER: Erlotinib-resistant H292 cells
HCC827 GR: Gefitinib-resistant HCC827 cells
HK1: Hexokinase 1
HK2: Hexokinase 2
NSCLC: Nonsmall cell lung cancer
OCR: Oxygen consumption rate
Osi: Osimertinib
OXPHOS: Oxidative phosphorylation
PDHA1: Pyruvate dehydrogenase E1-alpha subunit
Phen: Phenformin
PKM2: Pyruvate kinase isozymes M2
Ro: Rotenone
TKI: Tyrosine kinase inhibitor
TMRM: Tetramethylrhodamine methyl ester.

## Supplementary Materials

Supplementary Materials Supplemental Figure 1: (a) MA plot showing differential RNA expression levels in HCC827 GR versus HCC827 cells. Genes related to glycolysis were highlighted in red. (b) MA plot showing differential RNA expression in HCC827 and HCC827 GR cells. Genes related to OXPHOS subunits were highlighted in blue. Supplemental Figure 2: (a) Effect of rotenone, a representative complex 1 inhibitor, on cell proliferation of HCC827 and HCC827 GR cells. Both the cell types were exposed to 0.1 *μ*M rotenone and/or 1 mM AKB for 72 h, and cell proliferation was assessed by IncuCyte ZOOM. Data represent means ± S.E.M. (*n* = 6, ∗∗∗*P* < 0.001 vs. vehicle-treated group; ^#^*P* < 0.05, ^###^*P* < 0.001 vs. rotenone-treated group). (b) Effect of rotenone on mitochondria membrane potential. HCC827 and HCC827 GR cells were treated with rotenone for 1 h, and TMRM fluorescence was detected by flow cytometry. Data represent means ± S.E.M. (*n* = 3, ∗∗∗*P* < 0.001 vs. vehicle-treated HCC827 cells). (c) H292 and H292 ER cells were treated with vehicle or phenformin (30 and 100 *μ*M) for 72 h, and cell proliferation was analyzed with IncuCyte ZOOM. Data represent means ± S.E.M. (*n* = 6).
