# Supplementary material for: Enhanced Sensitivity of Nonsmall Cell Lung Cancer with Acquired Resistance to Epidermal Growth Factor Receptor-Tyrosine Kinase Inhibitors to Phenformin: The Roles of a Metabolic Shift to Oxidative Phosphorylation and Redox Balance
Source: Oxid Med Cell Longev. 2021 Jul 29;2021:5428364. doi: 10.1155/2021/5428364 (PMC8342158; doi:10.1155/2021/5428364)
Supplement: Supplementary Materials — Supplemental Figure 1: (a) MA plot showing differential RNA expression levels in HCC827 GR versus HCC827 cells. Genes related to glycolysis were highlighted in red. (b) MA plot showing differential RNA expression in HCC827 and HCC827 GR cells. Genes related to OXPHOS subunits were highlighted in blue. Supplemental Figure 2: (a) Effect of rotenone, a representative complex 1 inhibitor, on cell proliferation of HCC827 and HCC827 GR cells. Both the cell types were exposed to 0.1 μM rotenone and/or 1 mM AKB for 72 h, and cell proliferation was assessed by IncuCyte ZOOM. Data represent means ± S.E.M. (n = 6, ∗∗∗P < 0.001 vs. vehicle-treated group; #P < 0.05, ###P < 0.001 vs. rotenone-treated group). (b) Effect of rotenone on mitochondria membrane potential. HCC827 and HCC827 GR cells were treated with rotenone for 1 h, and TMRM fluorescence was detected by flow cytometry. Data represent means ± S.E.M. (n = 3, ∗∗∗P < 0.001 vs. vehicle-treated HCC827 cells). (c) H292 and H292 ER cells were treated with vehicle or phenformin (30 and 100 μM) for 72 h, and cell proliferation was analyzed with IncuCyte ZOOM. Data represent means ± S.E.M. (n = 6). [file 5428364.f1.zip › 5428364.f1.pdf]

Supplementary Figure 1

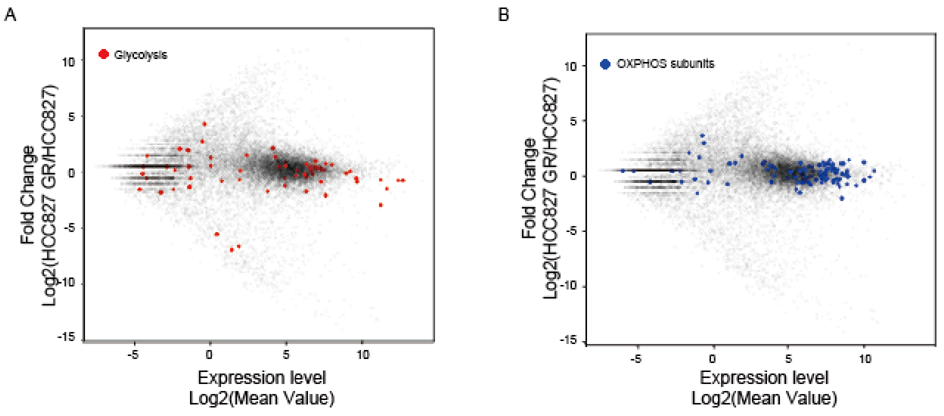

Supplemental Figure 1. (A) MA plot showing differential RNA expression levels in HCC827 GR versus HCC827 cells. Genes related to glycolysis were highlighted in red. (B) MA plot showing differential RNA expression in HCC827 and HCC827 GR cells. Genes related to OXPHOS subunits were highlighted in blue.

Supplementary Figure 2

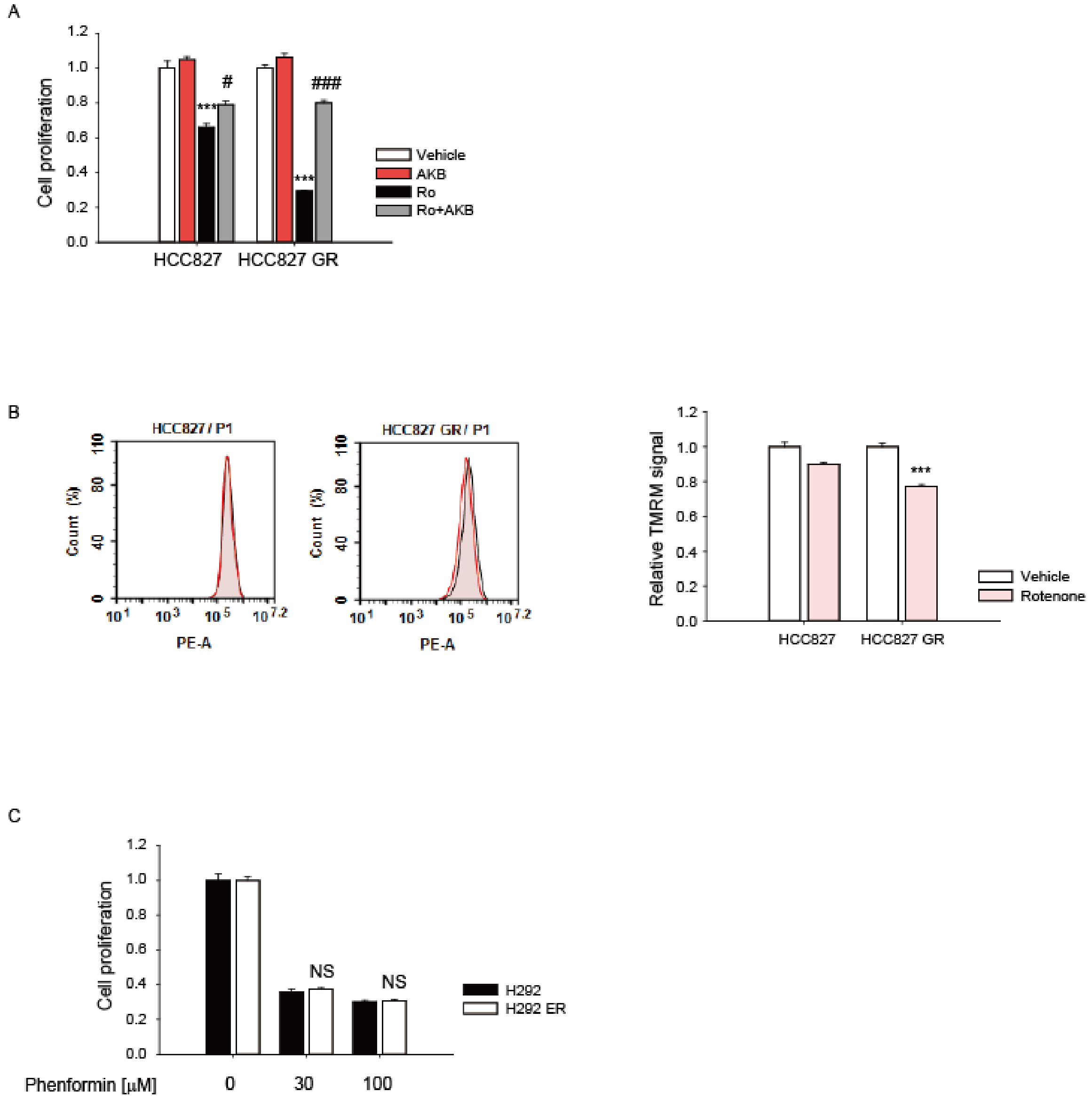

Supplemental Figure 2. (A) Effect of rotenone, a representative complex 1 inhibitor, on cell proliferation of HCC827 and HCC827 GR cells. Both the cell types were exposed to 0.1 μM rotenone and/or 1 mM AKB for 72 h, and cell proliferation was assessed by Incucyte Zoom. Data represent means ± S.E.M. (n = 6, $***P < 0.001$ vs vehicle-treated group; $^{\#}P < 0.05$, $^{\#\#\#}P < 0.001$ vs rotenone-treated group). (B) Effect of rotenone on mitochondria membrane potential. HCC827 and HCC827 GR cells were treated with rotenone for 1 h and TMRM fluorescence was detected by flow cytometry. Data represent means ± S.E.M. (n =3, $***P < 0.001$ vs vehicle-treated HCC827 cells). (C) H292 and H292 ER cells were treated

with vehicle or phenformin (30 and 100 μM) for 72 h, and cell proliferation was analyzed with Incucyte-Zoom. Data represent means ± S.E.M. (n =6).
